# Genetic and recombination analysis of GyVg1 varients from companion animals in central and northwest China

**DOI:** 10.3389/fvets.2025.1668033

**Published:** 2025-09-05

**Authors:** Zhibin Zhang, Xin Xu, Dandan Li, Fei Liu, Li Wang, Lunguang Yao, Jun Ji, Qingmei Xie, Yingzuo Bi

**Affiliations:** ^1^Henan Provincial Engineering Laboratory of Insects Bio-reactor, Henan Provincial Engineering, and Technology Center of Health Products for Livestock and Poultry, Henan Provincial Engineering and Technology Center of Animal Disease Diagnosis and Integrated Control, Nanyang Normal University, Nanyang, China; ^2^College of Animal Science, South China Agricultural University, Guangzhou, China

**Keywords:** phylogenetic analysis, antigenic epitope prediction, recombination analysis, mutation sites, cross-host transmission

## 1 Introduction

In 2021, the International Committee on Taxonomy of Viruses (ICTV) revised the classification criteria for several Gyrovirus species in the *Anelloviridae* family, renaming Avian gyrovirus 2 (AGV2) as Gyrovirus galga1 (1). GyVg1 is the second identified species within the Gyrovirus genus. However, its pathogenic mechanisms remain unclear, and its genetic characteristics are still poorly understood. The GyVg1 genome is a single-stranded circular DNA (ssDNA) molecule with a total length of ~2.37–2.38 kb, encoding three proteins via three open reading frames (ORFs) (2, 3). ORF1 encodes VP2, a scaffold protein that facilitates proper folding and capsid assembly of VP1, possesses phosphatase activity and regulates viral DNA replication (4). ORF2 encodes the VP3 protein, a non-structural protein functions as an apoptin, specifically inducing apoptosis in infected cells and suppressing the host immune response (5, 6). ORF3 encodes the VP1 protein, its N-terminal region of VP1 partially overlaps with the C-terminal region of VP2. VP1 is the major structural protein of the viral particle, forming an icosahedral capsid that protects the viral genome and serves as the primary target of the host immune system (7, 8).

In 2012, GyVg1 was detected in fecal samples from patients with unexplained diarrhea and in muscle samples intended for human consumption in Hong Kong (9). It was later found in both poultry feather shaft samples and human blood in mainland China, with high sequence similarity between strains (10). A study in South Africa also detected GyVg1 and related viruses in individuals with diarrhea and respiratory illnesses, as well as in healthy children (11). GyVg1 was first identified in 2011 in chickens with clinical symptoms in Brazil and has since been reported in poultry and poultry products worldwide (2). Retrospective analysis of frozen chicken meat samples in Japan revealed viral circulation since at least 1997 (12). Variant strains have been found in both symptomatic and asymptomatic chickens, showing 11.5–13.1% nucleotide divergence among distinct phylogenetic lineages (13). It has also been detected in poultry vaccines and in cases of co-infection with other avian pathogens such as Marek's Disease Virus (MDV), Newcastle Disease Virus (NDV), and Avian Reovirus (ARV) (8, 14–16). Recent studies have reported its presence in commercial chicken flocks in Vietnam, along with documented recombination events (17). Our previous report suggested that chicken-derived GyVg1 does not exhibit clear evolutionary or geographical distribution patterns (18).

More importantly, GyVg1 has been reported in multiple species. In 2019, it was detected in pet cats in northeastern China (19), and in fecal and tissue samples from farmed snakes in Hubei Province (20). In 2022, it was found in pet dogs, as well as in zoo animals including tigers, hippos, lions, sika deer, and various birds (21, 22). Evidence from 2020 to 2023 confirms its cross-species and geographical transmission potential (23). With increasing urbanization, companion animals such as pet cats and dogs play a growing role in human society. Given the frequent close contact between humans and pets, these animals may serve as reservoirs or intermediates for emerging viruses. Therefore, investigating the prevalence of GyVg1 in urban companion animals is critical for understanding its potential public health implications.

## 2 Material and methods

### 2.1 Sample processing and viral nucleic acid extraction

Between 2023 and 2025, routine disease screening for pet cats and dogs was conducted at veterinary hospitals from Henan, Shaanxi, and Gansu provinces. Serum samples were collected for viral nucleic acid extraction, with a total of 296 serum samples, including 137 from pet cats and 159 from pet dogs (details in Supplementary Table 1). To ensure ethical compliance, all pet owners provided informed consent, and sera collection protocol was approved by the South China Agricultural University Committee for Animal Experiments (Approval ID: SYXK 2019-0136, Approval Date: June 8, 2020). Viral nucleic acids were extracted using a commercial nucleic acid extraction kit, strictly following the manufacturer's instructions. The extracted nucleic acid was stored at −80 °C for subsequent experimental analysis.

### 2.2 GyVg1 screening and whole-genome sequencing

Conventional PCR amplification was performed using GyVg1-specific detection primers to screen positive samples. The specific primers used for detection were designed based on published literature (10). For PCR-positive samples, segmented amplification was carried out using PrimeStar HS DNA Polymerase (TaKaRa Bio Inc., Kusatsu, Japan) and three overlapping amplification primer sets designed in this study (sequences of detection and amplification primers are provided in Supplementary Table 2). The amplificons were cloned into the pMD-18T vector (TaKaRa Bio Inc., Kusatsu, Japan), and subsequently sequenced by Syn-Biotechnology (Suzhou, China). All experiments and sequencing procedures were repeated at least three times to ensure result accuracy.

### 2.3 Sequence similarity and phylogenetic analysis

The obtained DNA fragments were assembled using SeqMan software (DNASTAR, Lasergene^®^, Madison, Wisconsin) to generate the complete GyVg1 genome sequence. Similarity analysis was conducted using Bioinformatics Aider (v1.527) to compare the obtained GyVg1 genomes with reference strains from the NCBI database (24). Protein aa-sequence alignments were also performed to determine the genetic relationships among these strains. In total, 50 reference strains were selected for comparison, including two Human gyrovirus (HGyV) strains and 48 GyVg1s. Data visualization of sequence similarity was conducted using the online tool Chiplot (https://www.chiplot.online/). Sequence multiple-alignment was performed using the Clustal-W algorithm in MEGA 11 (Molecular Evolutionary Genetics Analysis, version 11.0.13; Pennsylvania State University, USA) (25). The phylogenetic tree was constructed using the maximum likelihood (ML) method with the optimal evolutionary model (HKY+G+I) and 1,000 bootstrap replicates. The final phylogenetic tree, generated in Newick (NWK) format using MEGA 11, was visualized and annotated using the Interactive Tree Of Life (iTOL) online tool (https://itol.embl.de/) (26).

### 2.4 GyVg1 recombination prediction

Recombination analysis of the obtained GyVg1 genomes was performed using Recombination Detection Program (RDP) v.4.8.3 with default parameters, incorporating seven recombination detection algorithms (MaxChi, BootScan, Chimera, 3Seq, GENECONV, SiScan, RDP). The identified recombination events were further validated using SimPlot software, and BootScan plots were generated. All final recombination results were visualized using Origin software (Version 2022, Origin Lab Corporation, Northampton, MA, USA).

### 2.5 Capsid protein epitope prediction and mutation analysis

Potential antigenic epitopes of the GyVg1 capsid protein (VP1) were predicted using DNAMAN 5.2.2 software. Mutation analysis was conducted by compiling the mutation sites of 19 GyVg1 strains and calculating the mutation frequencies at each site. The distribution of mutation frequencies across different sites was visualized using a rose diagram generated with the online tool Chiplot (https://www.chiplot.online/).

## 3 Descriptive results

**Figure 1:**
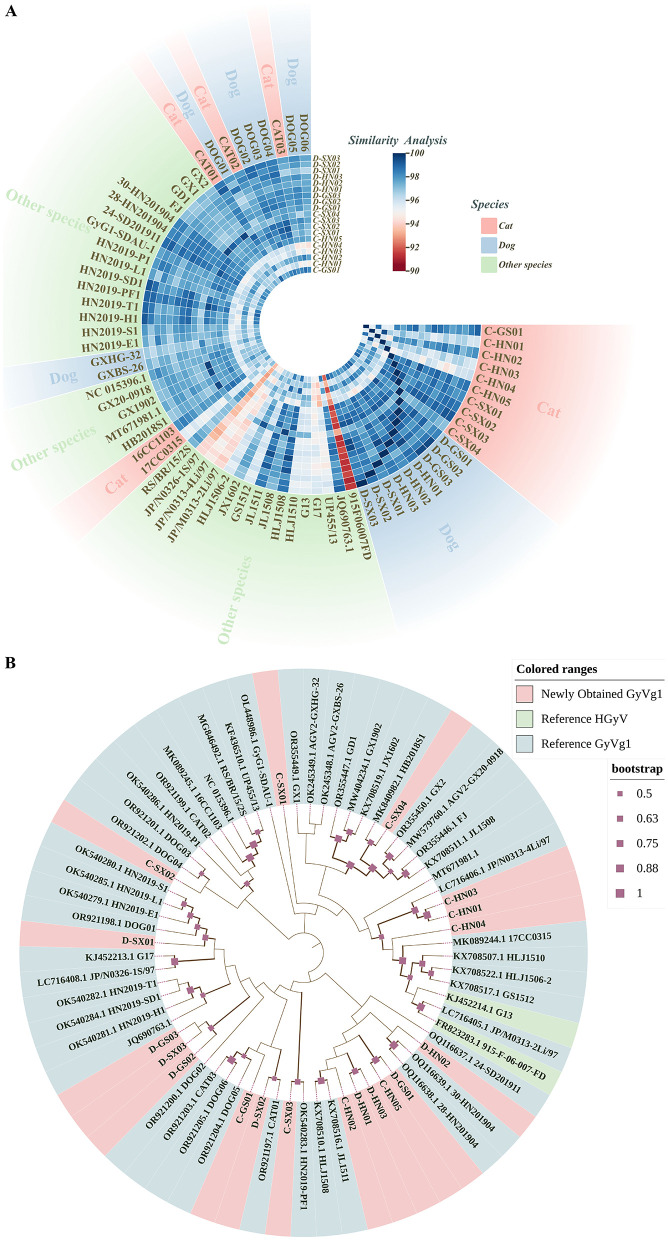
Comparative genomic analysis and phylogenetic relationships of GyVg1 strains. This figure provides an overview of the genetic relatedness among 19 GyVg1 strains identified in this study and 50 previously reported reference strains, based on whole-genome sequence comparison and phylogenetic inference. **(A)** Heatmap of whole-genome sequence similarity among the 19 newly identified GyVg1 strains and 50 reference strains. The gradient color scale represents pairwise genome similarity ranging from 90% to 100%. Outer color annotations indicate the host species (feline, canine, or others). **(B)** Whole-genome-based phylogenetic tree constructed using the maximum likelihood (ML) method under the HKY+G+I substitution model with 1,000 bootstrap replicates. Solid red squares along the branches denote bootstrap support values. Outer color annotations distinguish between the GyVg1 strains identified in this study and reference HGyV and GyVg1 strains.

Since its initial identification in 2011, GyVg1 has been detected in multiple hosts (21, 22, 27–29). Compared with avian-derived GyVg1, reports in mammals (e.g., cats, dogs, ferrets) remain limited. In this study, 19 GyVg1-positive samples were identified from 296 serum samples, with positivity rates of 7.30% in cats and 5.66% in dogs. These samples were distributed across Henan, Shaanxi, and Gansu provinces (Supplementary Table 3). The genomes of these 19 strains, all 2,376 nt in length, had been deposited to GenBank (accession numbers: PV941941-PV941959). Genome similarity among them ranged from 94.45% to 99.54%, and from 91.08% to 99.66% compared with 50 reference strains (Figure 1A). Phylogenetic analysis revealed that the 19 strains were scattered among different branches, while several ones were closely related to reference strains from peafowl, snakes and other avian species (Figure 1B).

**Figure 2:**
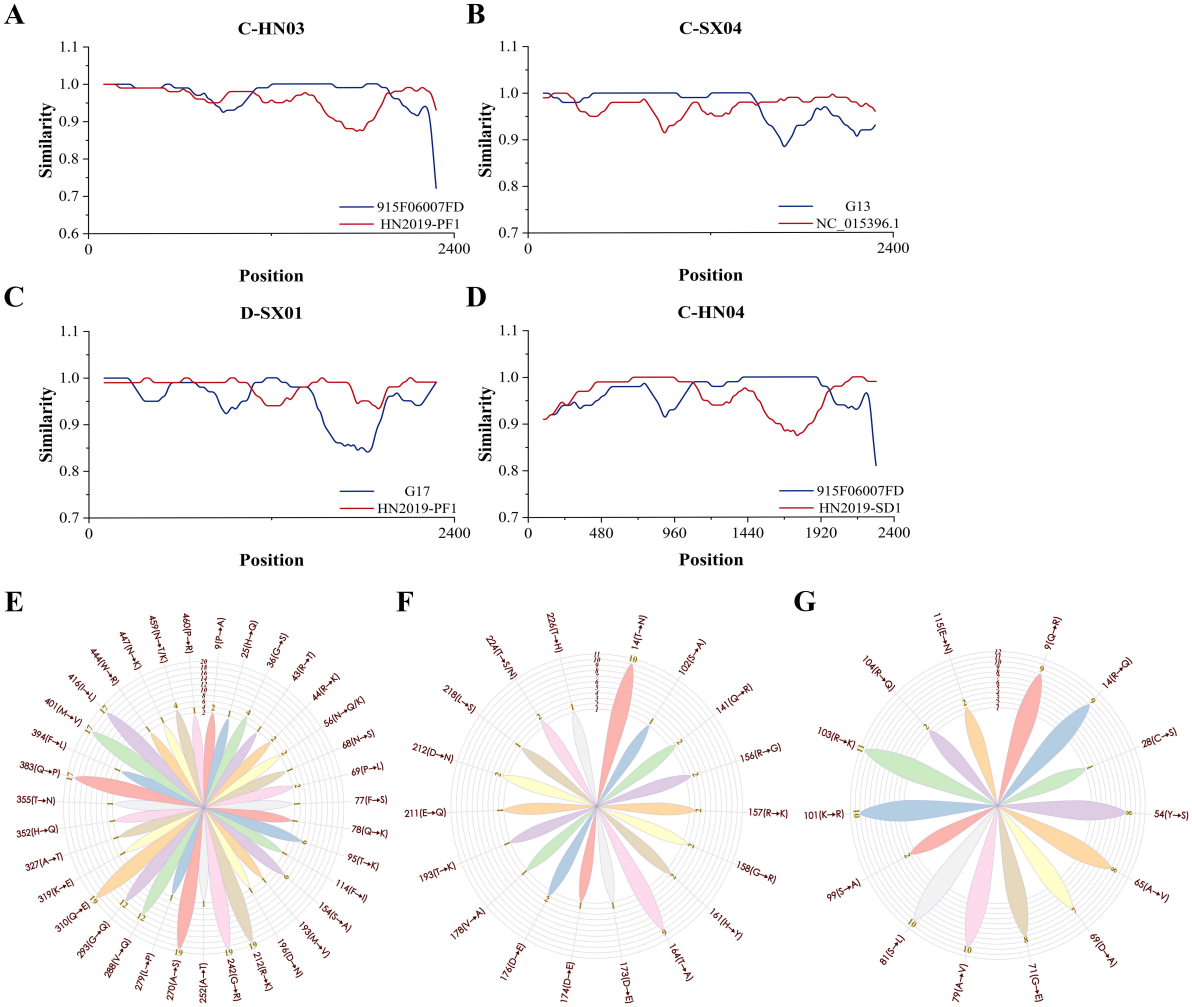
Recombination analysis and amino acid mutation profiling of 19 GyVg1 strains. This figure summarizes the recombination events and aa mutation characteristics observed in the 19 GyVg1 strains identified in this study. Recombination patterns were inferred using RDP4 and SimPlot, while aa mutation frequency and distribution were visualized across the VP1, VP2, and VP3 proteins. **(A–D)** Recombination events predicted using RDP4 and SimPlot software. **(A)** Strain C-HN03 was inferred to have resulted from recombination between 915F06007FD and HN2019-PF1. **(B)** C-SX04 was derived from recombination between G13 and NC_015396.1. **(C)** D-SX01 originated from recombination between G17 and HN2019-PF1. **(D)** C-HN04 was generated through recombination between 915F06007FD and HN2019-SD1. Recombination breakpoints are indicated by the intersection points of the plotted lines, marking potential recombinant regions. **(E–G)** Rose diagrams illustrating amino acid (aa) mutation sites and mutation frequency distributions in the coding regions of the 19 GyVg1 strains. **(E)** VP1, **(F)** VP2, and **(G)** VP3 panels show the positions of mutation sites, the corresponding aa substitutions, and their frequency distributions across the analyzed strains.

Recombination analysis using RDP4 and SimPlot predicted four recombination events (Figure 2, Supplementary Tables 4, 5). Events involved avian-, ferret-, and human-derived HGyV strains, suggesting that GyVg1 may evolve via recombination among diverse hosts. The involvement of human-derived HGyV in multiple events raised the possibility that humans could contribute to GyVg1 transmission, though this hypothesis required further investigation. A notable recombination event involved a peafowl strain (HN2019-PF1) detected from a zoo bird with limited environmental exposure, implying possible anthropogenic transmission. This suggested that GyVg1 might be exchanged among companion animals and captive birds in human-influenced environments. Given the close contact between pets and humans, pet-to-human or human-mediated transmission cannot be ruled out.

A total of 16 potential linear epitopes were predicted in the VP1 protein, with the region spanning residues 98–112 exhibiting the highest antigenic score (1.216), indicating its potential immunological significance (Supplementary Table 6). Amino acid (aa) variation analysis of the VP1, VP2, and VP3 proteins was performed using four reference strains: avian-derived GyVg1 (NC_015396.1), ferret-derived HGyV (G13), cat-derived GyVg1 (CAT01), and dog-derived GyVg1 (DOG01). For VP1, 36 substitution sites were identified, with nine (positions 212, 242, 270, 288, 293, 310, 383, 401, and 416) showing high mutation frequencies (≥12 strains). Among them, residues 212, 242, 270 and 310 were conserved across all 19 strains and the non-avian references, while site 459 showed divergent substitutions (N → T/K). In VP2 and VP3, host-specific mutations were observed: C-SX04 and D-HN02 shared several residues with the ferret-derived HGyV, in contrast to the avian, cat, and dog references; VP3 of C-SX04 was also particularly similar to HGyV. These patterns suggest that GyVg1 may undergo genetic adaptation to different hosts, potentially influenced by factors such as immune pressure and receptor specificity. Notably, 14 of the VP1 mutations were located within predicted antigenic epitopes, possibly affecting the virus's antigenicity and interaction with host immunity. However, their functional roles in host adaptation or immune evasion remain to be experimentally validated. Mutation site distributions are illustrated in the rose diagram (Figure 2).

To further investigate the potential transmission pathways of GyVg1, we conducted a survey of pet owners, which revealed these companion animals were exclusively fed commercial pet food and did not consume raw meat products. This feeding pattern indicates that foodborne exposure to GyVg1 through raw animal products is unlikely. Therefore, alternative transmission routes should be considered, including direct contact between companion animals, indirect environmental exposure, and human-mediated transmission through daily activities such as pet handling or cleaning of pet-related items.

This study conducted a genomic characterization of 19 GyVg1 strains derived from cats and dogs, revealing their genetic diversity, potential recombination events with strains from different hosts, and aa mutation patterns. Notably, these strains exhibited genetic similarity to a ferret-derived strain and showed comparable aa changes across the three coding proteins, suggesting complex evolutionary relationships with viruses from other hosts. These findings will provide valuable insights into the evolutionary mechanisms and cross-host transmission of GyVg1.

## Data Availability

The datasets presented in this study can be found in online repositories. The names of the repository/repositories and accession number(s) can be found in the article/Supplementary material.
