# Supplementary material for: Genetic and recombination analysis of GyVg1 varients from companion animals in central and northwest China
Source: Front Vet Sci. 2025 Sep 5;12:1668033. doi: 10.3389/fvets.2025.1668033 (PMC12446960; doi:10.3389/fvets.2025.1668033)
Supplement: Supplementary file 1 [file Data_Sheet_1.docx]

Supplementary Material

Genetic and Recombination Analysis of GyVg1 varients in Companion Animals in central and Northwest China China

Zhibin Zhang ^1^, Xin Xu ^1^, Dandan Li ^1^, Fei Liu^1^, Li Wang^1^, Lunguang Yao ^1^, Jun Ji ^1,*^, Qingmei Xie ^2^, Yingzuo Bi ^2^

**Correspondence:** Corresponding Author: [jijun020@126.com](mailto:jijun020@126.com)

**Supplementary Table 1.** Information on Collected Samples

| Province | Collected Samples | | | | Positive Samples | | | |
| --- | --- | --- | --- | --- | --- | --- | --- | --- |
|  | Pet Cats | Proportion of Total | Pet Dogs | Proportion of Total | Pet Cats | Proportion of Total | Pet Dogs | Proportion of Total |
| Henan | 59 | 19.93% | 62 | 20.95% | 5 | 1.69% | 3 | 1.01% |
| Shaanxi | 52 | 17.57% | 39 | 13.18% | 4 | 1.35% | 3 | 1.01% |
| Gansu | 26 | 8.78% | 58 | 19.59% | 1 | 0.34% | 3 | 1.01% |
| Total | 137 | 46.28% | 159 | 53.72% | 10 | 3.38% | 9 | 3.04% |

**Supplementary Table 2.** Detection and Amplification Primers for GyVg1

| Primer name | Sequences (5' to 3') |
| --- | --- |
| (detection) GyVg1-F | CGTGTCCGCCAGCAGAAAC |
| (detection) GyVg1-R | GGTAGAAGCCAAAGCGTCCAC |
| GyVg1-1F | ATTTCCTAGCACTCAAAAACCCATT |
| GyVg1-1R | TCTGGGCGTGCTCAATTCTGA |
| GyVg1-2F | TCACAGCCAATCAGAATTGAGCACG |
| GyVg1-2R | TTCTACGCGCATATCGAAATTTACC |
| GyVg1-3F | TATTCCCGGAGGGGTAAATTTCGAT |
| GyVg1-3R | CCCCTGTCCCCGTGATGGAATGTT |

**Supplementary Table 3.** Information on Collected GyVg1-Positive Samples

| Name | Age | Host | Accession no. | Year | Province |
| --- | --- | --- | --- | --- | --- |
| C-HN01 | 16m | Cat | PV941941 | 2023 | Henan |
| C-HN02 | 8m | Cat | PV941942 | 2023 | Henan |
| C-HN03 | 31m | Cat | PV941943 | 2024 | Henan |
| C-HN04 | 14m | Cat | PV941944 | 2024 | Henan |
| C-HN05 | 6m | Cat | PV941945 | 2025 | Henan |
| C-SX01 | 15m | Cat | PV941946 | 2023 | Shaanxi |
| C-SX02 | 10m | Cat | PV941947 | 2024 | Shaanxi |
| C-SX03 | 26m | Cat | PV941948 | 2024 | Shaanxi |
| C-SX04 | 10m | Cat | PV941949 | 2024 | Shaanxi |
| C-GS01 | 13m | Cat | PV941950 | 2024 | Gansu |
| D-HN01 | 8m | Dog | PV941951 | 2023 | Henan |
| D-HN02 | 12m | Dog | PV941952 | 2023 | Henan |
| D-HN03 | 29m | Dog | PV941953 | 2024 | Henan |
| D-SX01 | 9m | Dog | PV941954 | 2023 | Shaanxi |
| D-SX02 | 25m | Dog | PV941955 | 2024 | Shaanxi |
| D-SX03 | 7m | Dog | PV941956 | 2024 | Shaanxi |
| D-GS01 | 37m | Dog | PV941957 | 2023 | Gansu |
| D-GS02 | 24m | Dog | PV941958 | 2024 | Gansu |
| D-GS03 | 15m | Dog | PV941959 | 2025 | Gansu |

**Supplementary Table 4.** Recombination Events

| NO. | Recombinant  Sequence | Breakpoint Positions | | | Major Parent | | Minor Parent | | p-value |
| --- | --- | --- | --- | --- | --- | --- | --- | --- | --- |
|  |  | Begin | End | ORF |  | Similarity |  | Similarity |  |
| 1 | C-HN03 | 1106 | 1960 | VP1 | HN2019-PF1 | 96.09% | 915-F-06-007-FD | 93.56% | 1.14E-15 |
| 2 | C-SX04 | 1538 | 2349 | VP1 | G13 | 97.31% | NC 015396.1 | 97.18% | 5.12E-06 |
| 3 | D-SX01 | 1055 | 1346 | VP1 | HN2019-PF1 | 98.36% | G17 | 95.75% | 2.65E-05 |
| 4 | C-HN04 | 1068 | 1993 | VP1 | HN2019-SD1 | 96.3% | 915-F-06-007-FD | 92.76% | 3.84E-02 |

**Supplementary Table 5.**  Recombinant Events Predicted Using Different Algorithms

| Event | BootScan | Chimaera | GENECONV | MaxChi | RDP | SiScan | 3Seq |
| --- | --- | --- | --- | --- | --- | --- | --- |
| 1 | 1.96E-15 | 2.47E-13 | 1.12E-13 | 2.65E-13 | 1.14E-15 | 8.70E-16 | 8.55E-29 |
| 2 | NS | 4.11E-07 | 2.91E-04 | 5.11E-09 | 5.12E-06 | 1.02E-12 | 6.67E-15 |
| 3 | NS | 3.78E-02 | 3.31E-04 | 4.36E-02 | 2.65E-05 | 8.77E-03 | 1.77E-03 |
| 4 | 1.12E-02 | NS | 3.84E-02 | 3.26E-07 | NS | NS | 1.32E-14 |

**Supplementary Table 6.** Prediction of Capsid Protein Antigenic Epitopes

| Sites | Amino acid sequence (VP1) | Score |
| --- | --- | --- |
| 98-112 | NLTVCH**V**ASINVNLR | 1.216 |
| 422-433 | TPWC**V**VKVRSIW | 1.207 |
| 226-237 | FSPVA**S**LLVQND | 1.201 |
| 59-67 | PGSYV**V**RLP | 1.161 |
| 312-331 | SSRCFY**S**KACFPSFAALSAM | 1.155 |
| 173-180 | WALL**V**MHP | 1.135 |
| 72-93 | KLTLFF**Q**GIVFIPEAQAFVKST | 1.122 |
| 189-196 | APK**L**VTLD | 1.121 |
| 366-373 | LTLV**P**KGV | 1.113 |
| 388-408 | TDIAT**L**FLAQGSPVWAPYKFG | 1.111 |
| 132-143 | PYPQH**L**QGCQWS | 1.105 |
| 296-309 | SQ**N**VAPGIYRLAGL | 1.104 |
| 276-293 | EQWLPVNPPD**PP**VYPNQG | 1.095 |
| 207-220 | LF**R**HVKTKFRVLAT | 1.092 |
| 245-256 | E**G**FPVKGAPPMC | 1.088 |
| 162-168 | RPS**V**PPS | 1.067 |
